# Neuronal Dopamine D3 Receptors: Translational Implications for Preclinical Research and CNS Disorders

**DOI:** 10.3390/biom11010104

**Published:** 2021-01-14

**Authors:** Béla Kiss, István Laszlovszky, Balázs Krámos, András Visegrády, Amrita Bobok, György Lévay, Balázs Lendvai, Viktor Román

**Affiliations:** 1 Pharmacological and Drug Safety Research, Gedeon Richter Plc., 1103 Budapest, Hungary; b.kiss@richter.hu (B.K.); Visegrady@richter.hu (A.V.); Amrita@richter.hu (A.B.); Gy.Levay@richter.hu (G.L.); B.Lendvai@richter.hu (B.L.); 2 Medical Division, Gedeon Richter Plc., 1103 Budapest, Hungary; i.laszlovszky@richter.hu; 3 Spectroscopic Research Department, Gedeon Richter Plc., 1103 Budapest, Hungary; kramosb@richter.hu

**Keywords:** dopamine D_3_ receptor, localization, molecular structure, signalization, D_3_ ligands, dopamine D_3_ functions, therapeutic indications

## Abstract

Dopamine (DA), as one of the major neurotransmitters in the central nervous system (CNS) and periphery, exerts its actions through five types of receptors which belong to two major subfamilies such as D1-like (i.e., D1 and D5 receptors) and D2-like (i.e., D2, D3 and D4) receptors. Dopamine D3 receptor (D3R) was cloned 30 years ago, and its distribution in the CNS and in the periphery, molecular structure, cellular signaling mechanisms have been largely explored. Involvement of D3Rs has been recognized in several CNS functions such as movement control, cognition, learning, reward, emotional regulation and social behavior. D3Rs have become a promising target of drug research and great efforts have been made to obtain high affinity ligands (selective agonists, partial agonists and antagonists) in order to elucidate D3R functions. There has been a strong drive behind the efforts to find drug-like compounds with high affinity and selectivity and various functionality for D3Rs in the hope that they would have potential treatment options in CNS diseases such as schizophrenia, drug abuse, Parkinson’s disease, depression, and restless leg syndrome. In this review, we provide an overview and update of the major aspects of research related to D3Rs: distribution in the CNS and periphery, signaling and molecular properties, the status of ligands available for D3R research (agonists, antagonists and partial agonists), behavioral functions of D3Rs, the role in neural networks, and we provide a summary on how the D3R-related drug research has been translated to human therapy.

## 1. Introduction

The monoamine neurotransmitter dopamine (DA) is involved in several functions of the central nervous system (CNS) such as movement control, reward, feeding, olfaction, learning, cognition and in the peripheral nervous system (PNS) such as sympathetic, cardiovascular, renal and gastrointestinal [1], retinal [2], pancreatic functions [3] and in the immune system [4]. Diseases such as schizophrenia [5], Parkinson’s disease (PD) [6], attention deficit hyperactive disorder [7], depression [8], addiction [9], restless leg syndrome (RLS) [10] and pituitary tumors [11] are all related to the disbalance of the dopaminergic system.

Effects of DA are mediated through five receptor subtypes such as DA D_1_-, D_2_-, D_3_-, D_4_- and D_5_-receptors (D1R, D2R, D3R, D4R, D5R). All DA receptors belong to the G-protein coupled receptor (GPCR) family: D1R and D5R (D1-like family) stimulate cAMP signaling pathway through Gα_s_ G-proteins, whereas D2R, D3R- and D4R (D2-like family) inhibit this signalization through Gα_i/o_ G-proteins [12,13,14].

Cloning of the human D3R was first reported by Giros et al. in 1990 [15] which was followed by the cloning and characterization of the rat D3R [16,17]. Its structure, receptor properties including signaling pathways, in vitro and in vivo pharmacology, behavioral functions, potential involvement in various psychiatric (e.g., schizophrenia, drug abuse, depression, RLS) or neurodegenerative diseases are still under intense investigations.

The purpose of this review is to give an overview of our current knowledge and understanding on the molecular characteristics, the most prominent pharmacological properties, roles in behavior, and potential therapeutic utility of the neuronal D3R subtype.

## 2. D3Rs in the Brain and Periphery

**Figure 1:**
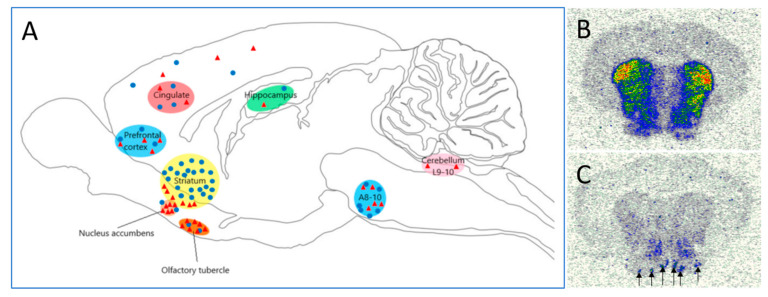
(**A**) D2Rs (blue circles) and D3Rs (red triangles) show distinct distribution within the rat brain (schematic drawing of the median sagittal section of a rat brain modified from the original image created by Gill Brown (source: neuroscience-graphicdesign.com, license: CC BY-NC 4.0). (**B**) [^3^H](+)PHNO binding in rat brain coronal section in the absence Gpp(NH)p visualizes both D2Rs and D3Rs. (**C**) [^3^H](+)PHNO binding in rat brain coronal section in the presence of 100 µM Gpp(NH)p allows visualization of only D3Rs. Arrows indicate the islands of Calleja, an area in the olfactory tubercle rich in D3Rs. The remaining binding dorsally to the islands of Calleja in the nucleus accumbens indicates high abundance of D3Rs in this area. Autoradiograms by F. Horti and B. Kiss (unpublished).

Membrane binding, autoradiography, mRNA hybridization and immunohistochemistry techniques revealed restricted expression/distribution of D3R in the rat brain and characteristically differed from the distribution of D2R [16,18,19,20,21] (Figure 1). The highest level of D3R expression was demonstrated in the islands of Calleja, ventromedial shell of nucleus accumbens, ventral tegmental area (VTA), substantia nigra (SN) and lobule 9, 10 of the cerebellum. D3Rs can transiently appear in layer 4 cells of the barrel cortex during the first 2 weeks of postnatal development. A distinct population of functional D3Rs was reported on pyramidal cells in layer 5 of the mouse prefrontal cortex. These type of D3R positive neurons mostly do not express D1R and D2R and have typical dendritic arborization, axonal projection pattern and electrophysiological profile [22,23]. D3Rs have been demonstrated to localize as somatodentritic autoreceptors in the VTA and SN, in granule cells of Calleja islands, and in the medium sized spiny neurons of accumbal rostral and ventromedullary cells [24,25]. Functional D3R were also demonstrated on rat hippocampal GABA-ergic neurons [26]. In other species, such as mouse, guinea pig and rabbit, similar to rats, the highest level of D3R was similarly found in the islands of Calleja, nucleus accumbens and in caudate nuclei, but out of the four species the mouse had the highest density in hippocampal D3R expression and the lowest in the frontal cortex [27,28,29]. In Rhesus monkeys D3Rs along with other DA receptor subtypes showed the strongest mRNA expression in layer 5 pyramidal neurons within the prefrontal cortex [30]. In addition, occurrence of D3Rs was reported in the superficial layer of the dorsal horn at the cervical and lumbar levels in the rat spinal cord [31].

In the *post-mortem* human brain a variety of techniques such as quantitative autoradiography, in situ mRNA hybridization and positron emission tomography (PET) were used to demonstrate regional distribution/expression of D3Rs. Receptor autoradiography studies have been performed with agonist ligands such as [^3^H]7-OH-DPAT) [32] or [^3^H]PD-128907 [33] or with antagonist ligand such as [^125^I]epidepride [34]. With in situ hybridization technique, D3R mRNA expression was found on principal cells of the prefrontal cortex in a laminated pattern. Abundant in basal ganglia, but low level of expression was also evident in cortical (i.e., anterior cingulate cortex) and subcortical regions (including anterior and medial thalamic nucleus, amygdala, mamillary body, SN pars compacta, locus coeruleus, raphe nuclei, lateral geniculate body, hippocampus, dentate gyrus and CA1 region) [35,36,37,38,39,40,41]. In contrast to the rat, in human no D3R mRNA expression in the VTA was reported [41].

The availability of the high affinity DA D_3_ receptor preferring agonist [^11^C]-(+)-PHNO [42] has made it possible to image and quantitate D3R in the living brain by PET. Although in the initial studies [^11^C]-(+)-PHNO was applied to demonstrate D2Rs of high affinity (D_2_^high^) in the rat brain [42,43,44], later, it also proved to be a highly useful ligand for imaging D3Rs in the rodent, primate and human living brain [44,45,46]. The highest level of D3R-PHNO binding was found in the SN-VTA (baboon), SN (mouse), globus pallidus (baboon and mouse) putamen and caudate (baboon) dorsal caudate (mouse) [45]. Human brain showed similarly high D3R binding in the globus pallidus, ventral striatum, putamen, caudate whereas the lowest binding was found in the cerebellum in each species [47]. Due to the low signal-to-noise ratio obtained in cortical regions, PET studies carried out with [^11^C]-(+)-PHNO did not report D3R-specific binding in human cerebral cortex. This technique, however provided great help to elucidate binding of antipsychotics to D3Rs in normal and schizophrenic subjects [48,49,50,51]. Combination of [^11^C]-(+)-PHNO PET imaging results with brain D3R and D2R mRNA expression (taken from postmortem transcriptome atlas), in accordance with previous findings, demonstrated highest level of [^11^C]-(+)-PHNO binding in the ventral pallidum, globus pallidus, nucleus accumbens and found strong correlation between [^11^C]-(+)-PHNO binding and D3R mRNA but not D2R mRNA expression [52].

D3Rs were demonstrated in peripheral organs such as the kidney [53] and by PET in the human retina [54]. In the human eye D3Rs were identified in the ciliary body epithelial cells where they heteromerized with melatonin-1 and melatonin-2 receptors [55]. D3Rs were found in the pancreas [56] and are thought to be involved in insulin secretion. The D3R was also identified in immune cells and evidence supports the involvement in immune responses [57,58,59,60]. The role/function of D3Rs in the peripheral organs or in the immune cells/response is largely unexplored.

## 3. Structure of Dopamine D3R

The D3R is a member of the G-protein coupled receptor family (GPCR). The largest phylogenetic class of GPCRs, known as class A, does not contain large extracellular domain only a transmembrane domain, with amino and carboxyl termini. Native ligands of aminergic GPCRs bind directly to the transmembrane domain, which is composed of seven transmembrane (TM) helices embedded in the cell membrane connected by three extracellular (EL) and three intracellular (IL) loops [61]. The C-terminus of the protein is the eighth small α-helix (H8).

The UniProt database [62] contains two described isoforms of the human D3R produced by alternative splicing and 1 potential isoform that is computationally mapped. The prevalent isoform is 400 amino acids long (UniProt ID: P35462-1) and contains a long ICL3 region. However, several additional alternatively spliced variants which do not bind DA have also been described in different species and are believed to function instead through the regulation of receptor dimerization [63].

Based on sequence analysis, the D3R shows higher similarity to the D2R (approximately 55% identity) than the D4R (approximately 40% identity) within the D2-like subgroup of the DA receptor subfamily (Table 1).

The main differences in the D2-like receptor sequences can be found mostly in the loop regions, especially in the IL3 between the TM5 and TM6. It is of note that the short and long isoforms of D2R or D3R also differ in this loop (UniProt sequences: P14416-1/P14416-2/P14416-3 and P35462-1/P35462-3, respectively). However, the differences in extracellular loop 2 (EL2) and in the extracellular side of the transmembrane region are of paramount importance for the development of D3R vs. D2R selective ligands.

There is only one experimental 3D structure of the hD3R available in the Protein Data Bank (PDB ID: 3PBL [65], which is in an antagonist binding (eticlopride) inactive conformational state of the receptor. In addition, a homology model of the hD3R in an active conformation is available in the GPCRdb (https://gpcrdb.org/), for which the 5-HT_1B_ receptor structure was used as main template (PDB ID: 6G79) [66]. Moreover, recent published X-ray and cryo-EM structures of other D2R-like receptors (e.g., PDB ID: 6CM4 [67], 6VMS [68], 6LUQ [69]) may be useful template structures for homology modeling. The suitable choice of template depends on the purpose of use. For instance, the risperidone-bound D2R structure (PDB ID: 6CM4) represents an inactive state which differs from the available D3R structure containing eticlopride [70]. Furthermore, the bromocriptine-bound D2R structure (PDB ID: 6VMS) is in an active state in complex with the G-protein.

**Figure 2:**
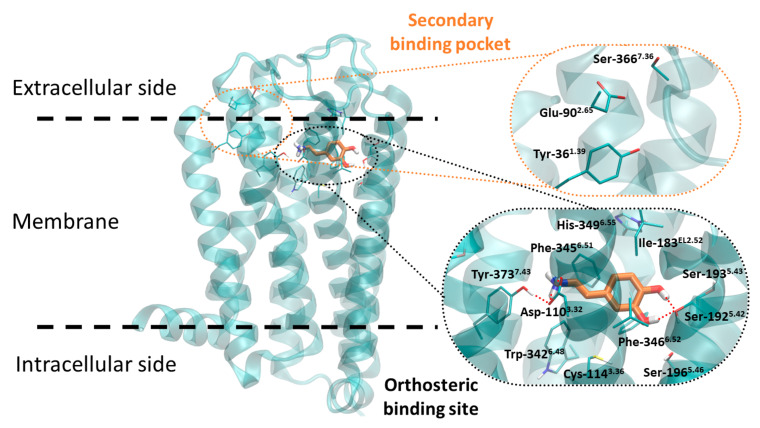
Structure of the hD3R, which contains the natural ligand (DA) docked into the crystal structure of D3R (ID is 3PBL in the Protein Data Bank; https://www.rcsb.org/).

Because of the structural similarity, a better understanding of the D3R may be supported by experience coming from aminergic GPCR research, and general structure-activity relationships may be applied to the D3R specifically. In general, there are several highly conserved residues in the orthosteric binding site (OBS) of aminergic GPCRs which form critical receptor-ligand interactions [71]. In the case of D3R (Figure 2) these are Asp-110^3.32^ (superscripts denote Ballesteros-Weinstein residue numbering [72]) forming a salt bridge with the cationic amine of the ligand, whereas Ser-192^5.42^ along with Ser-196^5.46^ interact with the meta-OH and para-OH moieties of DA. Furthermore, a cluster of aromatic residues in TM6 (Trp-342^6.48^, Phe-345^6.51^, and Phe-346^6.52^) interacts with the aromatic moiety of the ligand. Some residues in the EL2 are implicated in ligand specificity in aminergic receptors [73,74,75,76] especially Ile^EL2.52^, which is directly involved in ligand binding to the D2R [77]. This residue is the second residue after the conserved Cys in EL2 which forms a disulfide bond and can be defined as Cys^EL2.50^. The residue is analogous to the Ile-183^EL2.52^ in the D3R.

**Figure 3:**
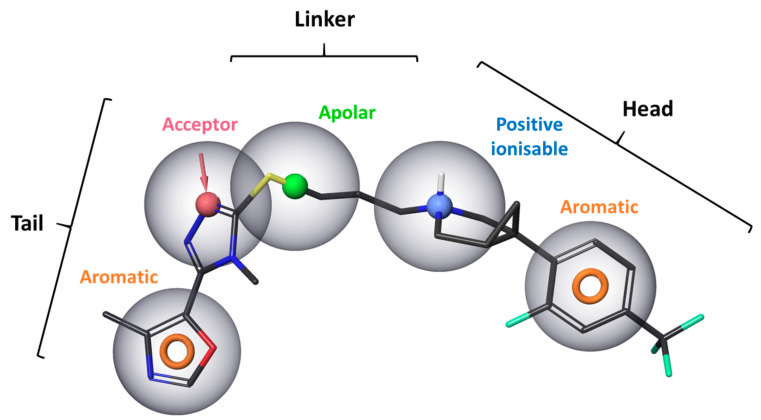
The D3R antagonist GSK-598809 and the general pharmacophore of D3R selective ligands.

Similar to other aminergic GPCRs, the D3R contains allosteric or secondary binding pockets which are less conserved than the OBS. OBS is highly homologous or even identical in a given subfamily or subgroups of aminergic GPCRs, which makes the development of subtype specific ligands extremely challenging. However, suggested by analyses of aminergic GPCRs, targeting the more divergent extracellular regions can be a possible way to achieve this. The solution can be ligands that bind concomitantly to the OBS and SBP, or allosteric modulators that bind only to SBPs, or compounds that differ in their interactions along entry/exit pathways to the OBS of different receptor subtypes [78]. The first approach is the most common strategy for development of D3R selective ligands. Most of the published D3R selective ligands are common in pharmacophore consisting of a head group, which is positively charged and bear an aromatic ring, and a heteroaromatic tail connected by an apolar linker region (Figure 3) [79]. This heteroaromatic tail which binds to the secondary binding pocket of the D3R is responsible for the high selectivity over the D2R (i.e., K_i,D2_/K_i,D3_ > 100) and can fine-tune the functional character of the ligands [80]. The linker may also be important in this regard [81].

The D3R, similarly to other GPCRs, possesses highly complex dynamics. Although experimental methods for studying GPCR structure and dynamics have advanced dramatically in recent years, a complete description of GPCR dynamics will require far more investigation. The D3R transmits a signal through the cell membrane via conformation rearrangement. Agonists bind to the extracellular side of receptor and favor structural changes that allow G-protein or arrestin to bind to its intracellular surface. Based on comparison of the available active and inactive GPCR structures, the most significant rearrangements occur in the intracellular site, especially in the TM6 region [82,83] and recently 13 state-specific conserved inter-TM interactions were identified [84]. It is possible to stimulate different intracellular signaling pathways independently via biased agonists [85,86] through a single GPCR. Many GPCRs possess secondary (allosteric) binding sites that influence intracellular signaling in distinct manners. The exact mechanism of biased and unbiased agonism is still unclear, but recent work suggests the significance of EL^2.52^, 5.43 and 6.55 residues (Ile-183, Ser-193 and His-349 in hD3R) in arrestin, and 5.42 (Ser-192 in hD3R) in G-protein biased signaling [87,88].

## 4. Signaling and Intracellular Pathways

D3R couples to G proteins in various cellular backgrounds, predominantly to inhibitory G_o_ proteins in recombinant systems [89,90] and although it displays high affinity towards DA, it also possesses lower signaling efficacy compared to D2Rs [91]. In addition to cAMP signaling, activation of MAPK kinase pathways and GIRK channels in vitro as well as the inhibition of P/Q-type calcium channels have been described downstream of D3Rs. Of note, both D3R-mediated inhibition of adenylyl-cyclase and stimulation of ERK phosphorylation have been suggested to involve Gβγ subunits [92,93,94]. 

In order to serve as transmitters of extracellular information, many G-protein-coupled receptors are subject to mechanisms for removal from cell surface or desensitization upon activation. Although PKC activation results in effective internalization of D3Rs from cell surface in various cell lines, DA induces only a marginal fraction of D3Rs to translocate to intracellular vesicles, in stark contrast to D2Rs [95,96]. Interestingly, a series of novel D3R agonists have been reported recently to induce D3R internalization independently of β-arrestins in vitro [97].

While internalization of D3R in general has not been reported, pharmacological sequestration, withdrawal of D3R into a more hydrophobic environment at the cell surface has been observed upon agonist-induced activation [96]. This β-arrestin-dependent process in recombinant systems does not seem to require GRK2 activation [96,97]. In addition to pharmacological sequestration, deubiquitinated β-arrestins play a role in Gβγ subunit binding and thus desensitization upon D3R activation [94]. Beyond their classical role in signal termination, little is known about β-arrestin-mediated signaling processes downstream of D3Rs. It seems not to involve ERK phosphorylation in heterologous expression systems [92] but a specific and more intricate pathway might exist in vivo. D3R-mediated regulation of Ca_v_3.2 calcium channels involves PKC pathway in cartwheel cells of the dorsal cochlear nucleus [22].

Based on proximity studies in heterologous expression systems, dimerization of D3Rs with various partners has been suggested. Moreover, both D1R-D3R and D3R-α4*β2 nAChR heteromers have been demonstrated on the cell surface of primary neurons [98,99] pointing at potential, yet unexplored regulatory mechanisms. Although the existence of an interplay in G protein signaling has not been ultimately dissected yet for D1R and D3R heteromers, cooperativity in MAPK activation and alteration of trafficking has been clearly demonstrated [99,100]. It is interesting nevertheless, that in the prefrontal cortex of mice little overlap in D1R and D3R expressing cells was found [23]. Finally, in addition to GPCRs, other receptor classes might also serve as heteromerization partners of DA receptors. The interaction of D3R with nicotinic acetylcholine receptors via β2 subunits has been implicated in neurotrophic and neuroprotective effects of nicotine at primary neurons in vitro [98,101].

## 5. D3R Ligands

Following the cloning of the rat and human D3R [15,16] a quest began for selective ligands to this new receptor subtype. Initially, various known DA receptor ligands and antipsychotics were systematically tested for D3R affinity and selectivity using recombinant rat and human receptors. In fact, several of these compounds displayed nanomolar or even subnanomolar affinity for D3Rs, but most of them demonstrated multi-receptorial profile with low D3R selectivity [16,17].

It was recognized soon after the discovery/cloning of the D3R that there exist significant similarities in molecular structure, signaling pathways and pharmacology between D2R and D3R, which greatly hindered the development of truly selective agonists or antagonists for the D3R. Availability of high affinity and selective ligands for D3R with good physicochemical and pharmacokinetic properties (i.e., “drug-likeness”) is indispensable for the understanding of the molecular biology, receptor interactions, signaling properties and in vivo functions of the D3R. The other big drive behind the quest for selective agonists, partial agonists or antagonists has been and still is the idea, that such compounds were thought to have great therapeutic potential in the treatment of various CNS diseases such as schizophrenia [16,102,103,104,105], drug abuse [9,106], depression [107,108] or PD [79,109]. 

As to the D3R ligands here we refer to the first, elaborate review by Levant (1997) [110], and by Gross and Drescher (2012) [103]. Extensive recent reviews have summarized the major developments in finding of new structures (agonists, partial agonists and antagonists), in vitro affinity and selectivity (versus D2R) of several D3R ligands [79,111,112,113]. An excellent review summarizing new lines in search for D2R/D3R drug research has recently been published [114]. Here, we point out some issues related to potency and selectivity and report on some most recent development in identification of selective agonists and antagonists.

### 5.1. High Affinity D3R Preferring Agonists

High number of agonists thought to be selective for the D3R have been described. However, sometimes it is difficult to assess the value and usefulness of the older and newly published agonists based on in vitro pharmacology. This is due to the fact that the reported binding affinities (i.e., inhibitor constant, Ki) and selectivity data and in vitro functional assessment coming from different laboratories (and data bases) are not always comparable, but sometimes may appear contradictory. These discrepancies were explained by the different assay conditions in different laboratories using different radioligands, cell lines and receptor species (human or rat). Many published agonists suffered from the lack of “drug-likeness” properties, i.e., they have unfavorable physico-chemical properties, insufficient absorption or low brain penetration, all these factors are greatly limiting the use for in vivo studies.

As an example, the published affinity and selectivity of the most frequently used D3R agonists PD-128907 [115] and 7-OH-DPAT [19] using membranes from various cells expressing human recombinant D3Rs varied in a wide range (e.g., D3R affinity of PD-128907 was reported between 0.4-18 nM, and its selectivity was between 6.1-100-fold range; whereas the same values for 7-OH-DPAT varied between 0.4–7.1 nM, and 31-100-fold range, respectively) [110,111,113]. A recent publication on a newly described D3R agonist, ML-417 (*vide infra*) convincingly illustrates the influence of different experimental conditions (e.g., buffer used, buffer composition and radioligands, [^3^H]methylspiperone or [^3^H]7-OH-DPAT) on in vitro binding affinity and selectivity. Differences may even depend on agonist structure, since the change of conditions has less impact on the affinity of the D3R agonist pramipexole [81]. 

PD-128907, 7-OH-DPAT, pramipexole, rotigotine, ropinirole are the most known D3R preferring D3R/D2R full agonists. Out of them, pramipexole, 7-OH-DPAT and PD-128907 are frequently used full agonists in in vivo D3R pharmacology research. Limitation to their use is that they produce biphasic dose responses in in vivo experiments. In low doses they elicit behavioral responses (e.g., hyposociability, yawning, penile erection, hypophagia) which are considered to be a consequence of D3R activation, and these behaviors can be inhibited by selective D3R antagonists. However, at higher doses, they cause hyperthermia, stereotypic reactions, stimulate locomotor activity which are all considered to be due to D2R agonism [115,116,117,118,119,120,121,122,123,124,125,126,127] (for further behavioral actions in Section 6). 

The D3R preferring D3R/D2R full agonists such as pramipexole, ropinirole and rotigotine have long been used for the treatment of motor disbalances of PD, RLS and depression (see Section 7). In vivo occupancy data are available for pramipexole only. It has been found to occupy D3Rs in rats [128] (ED_50_: 0.018 mg/kg, p.o.). Single dose of pramipexole (0.5 mg) was able to displace [^11^C]-(+)-PHNO binding in the D3R-rich globus pallidus, indicating the in vivo binding of this full agonist to D3Rs [48]. 

Discovery and characterization of ML-417 (compound *20*) has recently been published as an agonist of D3Rs with signaling routes involving ß-arrestin translocation, G-protein activation and ERK1/2 phosphorylation. Its affinity for D3R varied in a wide range (i.e., from 12.5 to 3700 nM) depending on the binding buffer composition (*vide supra*), but in the ß-arrestin functional assay it was 10,000-fold more selective for the D3R than for D2R. The compound demonstrated favorable pharmacokinetic and toxicology profile which may render it a good research tool [129]. 

### 5.2. High Affinity, Selective D3R Antagonists

Several compounds with diverse structures have been published as selective or highly preferring D3R antagonists with drug-like properties. Among those were U99194, GR 103,691, (+)-S14297 [130], SB-277011 [131], S33084 [132], RGH-1756 [133], ABT-127 [134], ABT-925 [135], RG-15 [136], GSK598809 [137], and PF-04363467 [138]. These compounds have been studied in vivo and produced behavioral effects which are thought to be related to their D3R antagonism (see Section 6). ABT-125, GSK598809 and S33084 were tested in humans and, indeed, achieved brain D3R occupancy but their clinical effects were mixed and unsatisfactory, thus their development was stopped in relatively early phase of human trials (see Section 7). SB-277011 and ABT-925 are frequently used as research tools. 

F17464 is a recently published high affinity, selective and potent D3R antagonist (Ki: 0.16 nM) with similar affinity but partial agonist activity at serotonin 5-HT_1A_ receptors (Ki: 0.16 nM) and 71-fold lower affinity for D2Rs (Ki: 12.5 nM). It demonstrated promising antipsychotic profile [139], occupied D3Rs in the human brain [140] and improved acute exacerbation of schizophrenia [141]. 

Some representatives of diazaspiro alkane [142] and 6-methoxy-1,2,3,4-tetrahydroquinolin-7-ol derivatives [143] showed high affinity (Ki < 20 nM) and high selectivity (D2R/D3R ratio: 200–1000) antagonists for D3R under in vitro conditions but the pharmacokinetic properties or their in vivo actions were not reported.

VK4-416 (compound 19) was described as potent and highly selective D3R antagonist (D3R Ki: 6.84 nM vs. D2R Ki: 11,400 nM) [144] and proved to be active in inhibiting oxycodone self-administration and reinstatement [145]. 

The stereoselective synthesis of (+)-VK04-87 and (−)-VK04-87, the enantiomers of (±)-VK04-87, a non-competitive allosteric antagonist at D3Rs [146] has been reported. The D3R binding affinity of (+)-VK04-87 was 17-times higher than that of (−)-enantiomer (Ki: 0.39 nM vs. 6.5 nM) and it was more potent and more selective in the antagonism of ß-arrestin recruitment assay (IC_50_ 55 nM vs. 1000 nM) than the (−)-enantiomer. No data were published on the pharmacokinetic and in vivo actions of the antagonists of this type [81].

### 5.3. Compounds with High Affinity for D3Rs with Various Functionality and Mixed Receptor Profile

A large number of antipsychotics has been tested for their affinity for D3Rs and in fact many of them displayed nanomolar or even subnanomolar affinity for D3Rs beside D1R, D2R, D4R and D5R, various serotonin, muscarinic and adrenergic receptors in the in vitro binding assays [147,148] (see also http://pdsp.cwru.edu/pdsp.asp). Despite several antipsychotics displayed high affinity for D3R in vitro, this property was not always translated to D3R occupancy as measured by [^3^H](+)-PHNO binding in the living rat [45,128,149,150] or human brain by PET studies with [^11^C]-(+)-PHNO [48,50,151]. The in vitro properties, behavioral effects, clinical actions and human D3R occupancy of D3R preferring D3R/D2R partial agonist antipsychotic cariprazine (subnanomolar affinity for both D3Rs and D2Rs, 5-8-fold selectivity for the D3R over the D2R) [152], blonanserin (subnanomolar but equal affinity for D3R and D2R receptors) [153,154], as well as the preclinical and clinical effects of the partial agonist BP-897 [155] have been reported (see Section 6 and Section 7). BP1.4979 is a high affinity (Ki: ~1 nM), low efficacy, potent partial agonist at D3Rs and antagonist at D2Rs with about 200-fold selectivity for D3Rs over the D2Rs, under development for smoking cessation and RLS. BP1.4979 has been shown to achieve high, dose dependent D3R occupancy in the human brain [156]. Among N-(4-(4-phenyl piperazin-1-yl)butyl)-4-(thiophen-3-yl)benzamides [157], MC-25-41 (D3R Ki: 0.5 nM, 1,486-fold selectivity over D2Rs) has been identified as potent and long acting partial agonist for the D3R. MC-25-41 has been found to reduce motivation for cocaine in rats and the long half-life of the drug may render it useful in the treatment of cocaine abuse [158].

### 5.4. D3R Imaging, PET Ligands

PET is a useful technique for receptor imaging and occupancy by investigational drugs in the living animal and human brain [159,160,161]. Due to the structural similarity of D3Rs and D2Rs, their similar localization in various brain areas (with higher density of D2Rs), the high affinity of DA for D3R, the in vivo imaging of D3R by PET have long been hindered by the lack of high affinity and selective PET ligands with good kinetic profile (i.e., low metabolic liability and good CNS penetration) for this receptor. Great efforts have been made to develop PET ligands (both agonists or antagonists) for in vivo imaging of D3Rs and D2Rs. Many of them displayed high affinity for both D2Rs and D3Rs, but either their selectivity or physicochemical properties or brain penetration were far from satisfactory [162,163,164,165,166] to differentiate between imaging of D2Rs and D3Rs. Availability of the high affinity and D3R preferring agonist [^11^C]-(+)-PHNO [42] has made possible to image D3Rs in the human brain [44,167].

## 6. Behavioral Functions of the D3Rs

The D3R-system is generally accepted to be involved in the regulation of cognitive, social, emotional, motivational and locomotor processes [104,168]. In this chapter we attempt to review the behavioral pharmacology of D3R with respect to cognition, social-emotional behavior and locomotion. 

In the first instance, the implication of the D3Rs in the above aspects of behaviors was based on brain-wide expression studies [18,29] indicating specific localization in the neuro-substrates of these behaviors, namely limbic and cortical structures (Figure 1). A more in-depth, experimental investigation of the D3R-system was made possible by the advent of acceptably selective agonists such as 7-OH-DPAT, pramipexole or PD-128907 and antagonists such as SB-277011 or ABT-925 [19,102,115,131,169]. Although under in vitro conditions these pharmacological tools appear to be satisfactorily selective for D3R versus D2R, selectivity of agonists especially greatly diminishes under in vivo conditions [124,170] (see also in Section 3). Whether D3Rs are indeed functionally active in vivo was demonstrated by exploiting selective antagonism and pharmaco-MRI technology in rats [171]. The role of D3Rs in cognitive and emotional processes was further bolstered by using classical behavioral pharmacological methods [104,168]. In addition to traditional behavioral pharmacology approaches, genetically modified animals including mice lacking or overexpressing the D3Rs have also contributed to a better understanding of the neurobiology of the D3R-system [172,173,174].

### 6.1. Role in Locomotor Activity

While the dopaminergic system en bloc has long been known to be centrally involved in the regulation of movement, the role that D3Rs play in particular is still a matter of controversy. Without doubt, the regulation of locomotor behavior has an intricate foundation, yet beyond the complexity of this biological process, the blurred picture about the involvement of D3R is mostly due to the less than optimal in vivo selectivity of tool compounds (both agonists and antagonists) targeting these receptors. The limited nature of in vivo selectivity for the D3Rs is perhaps most tangibly illustrated by the finding that D3 preferring agonists such as 7-OH-DPAT and PD-128907 cause a biphasic ambulation response, with hypomotility at low doses and hypermotility at higher doses [120,125,126,127]. Since D3R agonist-induced hypomotility can be reversed by selective D3R antagonists [125], but not by selective D2R antagonists [126], this response is considered D3R specific. The hypermotility induced by higher doses is a D2R-like effect, as it can be blocked by D2R preferring antagonists such as L741,626 and S23199 [126]. Initially, D3R antagonists (nafadotride, U99194) were found to induce hypermotility when administered alone [169,175]. Surprisingly, more selective antagonists (SB-277011, S33084, GR218231) did not alter locomotor activity at their D3-selective doses in novel environments [131,132]. In contrast, when D3R antagonists were investigated in animals habituated to recording conditions, a marked hyperactivity response was found possibly due to D3R-mediated, confounding attentional and emotional modulation of locomotor activity [125]. This being seemingly settled, the picture is further complicated by the facts that (1) lack of the D3R does not necessarily lead to changes in motor activity [176], (2) D3R agonists do not cause hypolocomotion in D2R knockout mice [177] and (3) the D3R antagonist U99194 elicits hyperactivity in D3R knockout mice [178]. Based on all the above, it seems that such a simple rule that D2Rs increase, while D3Rs reduce motor activity cannot be endorsed. Revealing the exact role of D3Rs in the regulation of locomotion may depend on the generation of in vivo truly selective D3R agonist compounds, conditional knockouts or the employment of optogenetic approaches.

### 6.2. Role in Learning and Cognition

The first idea that D3Rs play a role in learning and cognitive processes based on brain localization of the receptors was further supported by findings with selective pharmacological agents. As a rule of thumb, increasing D3R function (e.g., agonism, increased receptor expression) generally impairs, while reducing D3R function (e.g., antagonism, lack of the receptor) improves cognitive and mnemonic performance [179,180]. The role of the D3R in cognition has been extensively reviewed earlier [102,168,181].

Pharmacological stimulation of the D3Rs by systemic exposure to 7-OH-DPAT or PD-128907 impairs various elements of cognitive functioning (e.g., working memory, attention, processing speed and executive function) from rodents [182,183,184,185] to nonhuman primates [186,187]. Activation of the D3Rs by pramipexole may cause cognitive adverse events even in humans [188]. Compromised cognitive performance upon D3R activation is associated with reduced regional cerebral blood flow in prefrontal and limbic cortical regions in baboons after pramipexole treatment [189]. On the neurophysiological level, D3R agonism may lead to decreased synchronized electrophysiological activities necessary for proper cognitive functioning [190]. Furthermore, increased D3R expression was found in the prefrontal cortex in rats in a neurodevelopmental model of schizophrenia induced by gestational methylazoxymethanol acetate exposure that recapitulates cognitive impairments typical of the disorder [191]. D3R blockade can reverse the behavioral effects of NMDA receptor blockade [192] in acute psychosis models, such as NMDA receptor blockade by ketamine or phencyclidine that also produce cognitive deficits in rodents and exacerbate D3R function.

In contrast to D3R stimulation that impairs cognition, systemic D3R blockade by selective antagonists (e.g., SB-277011, RGH-1756, S33084) or D3R preferring, selective D3R/D2R antagonists (e.g., S33138, U99194, RG-15), improves general or social cognitive deficiencies inflicted by various manipulations in rodents (Table 2 and Table 3) [102,179,182,185,193,194,195,196,197,198] with a few exceptions [193,199]. In case of selective or highly preferring D3R antagonists (ABT-925, ABT-127, GSK598809) that have been characterized in detail in in vitro studies and other behavioral models (e.g., drug addiction models), public data on pro-cognitive effects in preclinical models is not available in most cases or scarce at best such as for the D3R preferring antagonist F17464 [139,200]. Data on D3R related cognitive effects in non-human primates is also scarce. A study by Millan et al. (2010) [201] shows that the D3R preferring antagonist S33138 improves cognitive performance in Rhesus monkeys.

Not only full antagonists (agents without intrinsic activity), but also compounds that are D3R preferring partial agonists (functioning as antagonists under high dopaminergic tone) such as BP-897, cariprazine, or its metabolite, didesmethyl-cariprazine also improve cognitive deficits in various animal models [150,194,204,205,206] (Table 4). In case of cariprazine this pro-cognitive action translates well into humans, as it improved cognition in schizophrenic patients, which is not typical to antipsychotics [207]. ABT-925—the only selective D3R antagonist that entered clinical testing in schizophrenia—is another example for pro-cognitive efficacy in humans. ABT-925 was ineffective on its primary endpoint [208] nevertheless, it resulted in signals for improvement in executive function (verbal recognition memory task) and emotion recognition in a subset of patients [102] (see also in Section 9). 

The specific role of D3R antagonism in cognition is evident in D3R receptor knockout mice, where compounds with a D3R component in their molecular profile such as buspirone or cariprazine are unable to reverse MK-801 or PCP-induced cognitive deficits [209,211]. Similarly, the D3R antagonist Y-QA31 could not reverse a MK-801-induced cognitive deficit in D3R knockout mice [197]. Loss of D3Rs due to genetic manipulation leads to cognitive improvements similar to systemic treatment with D3R antagonists [172,173,185,212] accompanied by increased striatal and accumbal DA levels [213,214] as well as cortical neuronal activation. The lack of D3R can also prevent age-related spatial memory decline in mice [215]. Hypofunction of the D3R concomitant with reduced dysbindin-1 expression rescues working memory deficits inflicted by heterozygous knockout of the dysbindin-1 gene [216]. Not all reports are in favor of a pro-cognitive effect of knocking out the D3R as this condition in mice did not alter T-maze working memory function in a study by Chourbaji et al. [217]. On the other hand, an excess of D3Rs limited to the striatum does not necessarily cause cognitive deficits in mice as could be expected theoretically based on studies with D3R agonists but disrupts motivation [174].

### 6.3. Role in Emotional Regulation

Just like in case of locomotor behavior discussed above, the exact role of D3Rs in emotional regulation (anxiety, motivation and behavioral stress reactivity) is not completely understood (Table 5). Studies in D3R knockout mice have delivered equivocal evidence for the function of D3Rs in the regulation of emotionality; there are studies showing reduced [218,219], increased [220] or unchanged levels of anxiety or depressive-like behaviors [217,221] in mice lacking D3Rs.

Literature data about anxiolytic or anti-anhedonic effects of D3R ligands is scarce and also somewhat contradictory. Prototypical D3R preferring antagonists with D2 receptorial actions, such as nafadotride and U99194, produced anxiolytic-like effect in animal models [222]. Microinjection of U99194 into the basolateral amygdala decreased anxiety-like behavior [223], yet the same compound given systemically did not reverse anxiety induced by chronic cannabinoid exposure in adolescent rats [203]. 

There are more data available on D3R agonists and partial agonists, showing an unequivocal picture for behavioral effects. D3R agonists such as 7-OH-DPAT, ropinirole or S32504 were anxiolytic and antidepressant-like in various species (mouse, rat and marmoset) and models [127,224,225,226]. Likewise, the D3R partial agonist BP-897 induced anxiolytic-like effects in the elevated plus-maze and Vogel punished drinking tests however, it was inactive in the forced swimming test [227,228]. The partial agonist cariprazine also reduced anxiety in the novelty-induced hypophagia test [229]. With respect to anhedonia, cariprazine reduced chronic stress-induced anhedonia both in rats [230] and mice [229]. Neither anxiolytic nor anti-anhedonic effect of cariprazine was found in D3R knockout mice [229], which indicates that partial activation of the D3R specifically may be anxiolytic and antidepressant.

### 6.4. Role in Social Behavior

A number of studies summarized in Table 2 show that inhibition of the D3R improves social recognition memory [179,182,185]. However, beyond effects on memory components of social behavior, D3Rs also seem to play a role in regulating other aspects of social behavior (Table 6). When administered systemically, D3R agonists have been shown to reduce huddling in rats [117] and social interaction in mice [231]. Though, D3R agonists are not necessarily detrimental to social behavior when applied locally into the lateral septum [232]. PD-128907-induced inhibition of huddling [117] has been even used as pharmacodynamic assay in drug development and contributed to the identification of the D3R antagonist A-690344 that suspended huddling deficits produced by the agonist [233]. The D3R antagonists ABT-127 and ABT-925 are further examples that prevented PD-128907-evoked inhibition of social behavior [234,235]. Furthermore, the administration of D3R antagonists such as U99194, increased social interaction and reduced aggression in single-housed mice [236]. U99194 restores social interaction deficits induced by PCP in mice which effect is counteracted by the D3 agonist 7-OH-DPAT [154]. Similarly, the D3R selective antagonist F17141 reverses subacute MK-801-induced social interaction deficits in mice [192]. The D3R full antagonist F17464 counteracted the social deficit in an animal model of autism caused by prenatal valproate exposure [140,237]. The D3R partial agonist cariprazine is also able to facilitate social interactions in animal models of schizophrenia [205,209,210]. 

## 7. Site and Mechanism of Action for D3R Antagonists

In order to adequately perform cognitive and social behaviors required by the environment, proper functioning of the prefrontal cortex is indispensable [237,238,239]. It only follows that malfunctioning of the prefrontal cortex may very well be in the background of negative and cognitive symptoms of psychiatric disorders such as schizophrenia or depression [240]. Although the prefrontal cortex expresses low levels of D3R [37], these receptors seem to be important players in frontocortical processes [168]. The exact mechanism whereby D3R ligands influence frontocortical functions is not known nevertheless, there are two potential modes of action for them: (1) indirect effect on prefrontal DA receptors by modulation of prefrontal DA release through presynaptic D3 autoreceptors in the VTA and SN or (2) direct action through postsynaptic D3Rs in the prefrontal cortical areas.

### 7.1. Antagonism of Presynaptic D3 Autoreceptors: DA Release in the Forebrain

The somatic D3 autoreceptors inhibit DA release in the nucleus accumbens or prefrontal cortex, the projection fields of the VTA and SN dopaminergic neurons [24,241]. According to the prevailing DA hypothesis of schizophrenia, reduced frontocortical dopaminergic tone resulting from excessive D2 and D3 autoreceptor stimulation in the midbrain due to striatal hyperdopaminergia is key to negative and cognitive symptoms of the disorder [5]. Consequently, social or cognitive dysfunctions unfolding after treatment with D3R agonists in experimental animals may represent negative symptomatic models with a firm mechanistic basis [102,242]. In line with this, the D3R agonist PD-128907 reduces the frontal release of DA and the antagonist S33084 can reverse this neurochemical alteration [201] parallel to its pro-social and pro-cognitive effects [179,182,185]. Other D3R antagonists including ABT-925, ABT-127, S14297, S33138 and SB-277011 also disinhibit the activity of VTA and SN DA neurons and thereby increase extracellular DA levels in the prefrontal cortex [132,234,241,243]. Some compounds can increase basal DA release in the prefrontal cortex and striatum such as the D3R preferring D2/D3 antagonist IRL-790 also known as mesodopetam [244]. The D3R preferring partial agonist cariprazine increases DA release in the nucleus accumbens and ventral hippocampus [245] as well as DA turnover in the striatum [204] in intact animals. Cariprazine also increases DA release in the prefrontal cortex in a PCP-exposure model of acute psychosis [246].

### 7.2. Indirect Effect through D1R

Antagonism at D3 autoreceptors thus brings about increased prefrontal DA levels. Elevated DA in turn may stimulate cortical D1R which can result in pro-cognitive and pro-social behavioral changes. The cognitive enhancing effect of D1R agonism is quite well established since activation of D1Rs by selective full agonists such as SKF81297 or A77636 improves various aspects of cognitive performance in rodents as well as primates [247,248]. Similar to many other cognitive enhancers, D1R agonists affect cognitive performance according to an inverted U-shaped dose-response curve [247,249].

Conversely, reduction of D1R expression and function in the prefrontal cortex due to chronic social defeat stress in mice is accompanied by social interaction deficits [250]. The specific role of prefrontal D1R receptors in cognition was demonstrated in rats with working memory deficits induced by local GABAergic inhibition of the prefrontal cortex [251]. Beside cognitive effects, the D1Rs seem to be involved in the regulation of social behaviors too. Social interaction was found compromised in a rat model where D1Rs are mutated in a way that insertion of the receptors is less likely in the neuronal membranes [252]. In another genetic mouse model of ASD, impaired social behavior was normalized by treatment with the selective D1R agonist SKF38393 [253]. 

Although an indirect activation of the D1Rs due to D3R antagonism and forebrain DA release seems to be logical, this issue has to be assessed critically. The D1R shows the least affinity for DA with a Ki value of approximately 1500–2000 nM [254]. In vivo microdialysis studies indicate that forebrain DA levels in the extracellular space (under baseline conditions or even after pharmacological manipulations) are in the pico-to-nanomolar range [243,245,246] which seems to be far below the concentrations that would be needed for substantial D1R binding and activation. On the other hand, transmitter levels determined by microdialysis indicate only extracellular presence but not intrasynaptic DA exposure which is the mediator of synaptic transmission. Voltametric studies in living animals or in slice preparations indicate that electric or optogenetic stimulation of afferents can result in synaptic DA overspill reaching concentrations of several hundreds of nanomoles to 1 micromole [255,256,257,258]. In extreme cases, such as in methamphetamine-sensitized animals, evoked synaptic DA release can reach levels exceeding 3 micromoles in the striatum, nucleus accumbens or ventral pallidum [259]. Yet, it is still a question whether under in vivo conditions, DA in the synapse due to somatic D3R antagonism can reach levels that indeed may activate D1Rs?

### 7.3. Indirect Effect through D2Rs

Theoretically, the D2R may also be considered as target receptor for increased dopaminergic transmission in the prefrontal cortex. However, in case of D2R activation, results are not unequivocally in favor of cognitive enhancement. Systemic D2R stimulation by the selective full agonist sumanirole improves cognitive performance in monkeys [260] which may be partly or completely due to the anxiolytic effect of the drug [261]. Studies with the less selective D2R agonist quinpirole delivered evidence that support a pro-cognitive role for the D2R [180] although there are also results to the contrary [262]. Therefore, indirect D2R activation due to increased prefrontal DA release is not a likely explanation for D3R antagonist effects on cognition.

### 7.4. Direct Effect of D3R Antagonists through Prefrontal D3Rs

In addition, or—most likely—alternative to an indirect effect through prefrontal D1 receptors, direct inhibition of postsynaptic D3Rs specifically in the same area possibly plays an important and central role in the pro-cognitive and pro-social effects of D3R antagonists. Antagonism of D3Rs in the prefrontal cortex leads to social or object recognition memory improvements, while antagonizing these receptors restrictively in the nucleus accumbens or the striatum does not have such effects [179,185]. Congruently, overexpression of D3Rs in the striatum does not result in cognitive dysfuntions [174]. Since a number of D3R antagonists also have affinity for the D2Rs and antagonistic effects may appear with higher dosing, antagonism through cortical D2Rs emerges as an alternative, although very unlikely mechanism for behavioral effects. Indeed, this does not seem to be the case as the preferential D2R antagonist L741626 given directly into the frontal cortex in rats did not enhance social recognition memory [179].

Recent advances have started to shed light on the intricate signal transduction pathways influenced by the D3R. Beyond the well-known negative regulation of adenylyl cyclase, the D3R is involved in other, G-protein-independent intracellular signaling processes (e.g., GRK, ß-arrestin, GSK3, Akt, etc., see also in Section 5). Exposure to novelty such as in the novel object recognition task, is associated with the dephosphorylation of the two GSK3 kinase isoforms in the medial prefrontal cortex of mice [202]. GSK3 dephosphorylation is practically eliminated in DA transporter knockdown mice that also show deficits in novel object recognition performance, both of which can be rescued either by the genetic knockout of the D3R or the administration of the D3R antagonist FAUC365 [202]. Conversely, stimulation of D3Rs by the D2R/D3R agonist quinelorane causes a rapid and transient increase in the phosphorylation of GSK3, Akt and mTORC1 effectors in the nucleus accumbens and dorsal striatum in rats in vivo which can be prevented by the administration of the D3R antagonist S33084 [263]. In vivo administration of quinelorane does not lead to GSK/Akt phosphorylation in mice lacking the D3R [263]. Given the involvement of GSK/Akt signaling in cognitive processes [264] this signal transduction pathway may be a means of cognitive and possibly socio-emotional effects of D3R antagonists. This notion has encouraged drug research efforts aiming at developing agents with dual D3R partial agonist and GSK3β kinase inhibitor profile [265].

## 8. D3R Function in Active Neural Networks

To understand the link between receptorial actions and in vivo behavioral responses we need to know the system-level alterations evoked by D3R-selective ligands in the CNS. Here, we list the potential points of interventions for D3Rs in active neural processes and make an attempt to explain some of the characteristics of these effects.

### 8.1. D3Rs in Gamma Oscillatory Activity

D3Rs have been implicated in the alteration of hippocampal gamma oscillations in acute brain slices [266]. In general, oscillatory activity integrates neural networks within and across brain structures during cognitive processes, therefore the changes in oscillation power reflects changes in cognitive functions. Gamma oscillation is a measure of synchronous activity at frequencies between 30 and 90 Hz [267] and is shown to be generated by fast-spiking parvalbumin-containing perisomatic interneurons [268]. Brain oscillations at the gamma band serve as a mechanism that brings a widely distributed set of neurons together into a coherent ensemble that is believed to be one underlying process when antipsychotics improve behavior in rat models of schizophrenia [269]. The power of gamma oscillations correlates with spatial recognition memory in mice [270]. In humans, alteration of gamma activity correlates well with sensory processing, perception, attention, working memory or long-term memory representation [271,272]. Disturbed cortical gamma band oscillations have been observed in schizophrenia [273,274] in a unique divergence; while positive symptoms of the disease (e.g., in patients with hallucinations) correlate with an increase in gamma oscillation power [275,276], reduced gamma is associated better with the negative symptoms of the disease [277]. In accordance with the theories of generation, the abnormalities in oscillatory activity in schizophrenia are believed to connect to alterations in GABAergic function [273,274]. The D3R-preferring partial agonist cariprazine could improve the periodicity of moderate and unsaturated gamma activity while it stabilized the power of gamma oscillations altered by MK-801 treatment in rat hippocampal slices suggesting a benefit arising from the partial agonistic character on D3R by cariprazine [278]. 

### 8.2. Rare Expression of D3R Requires More Efficient Intrinsic Receptor Function

One significant concern regarding brain functions of D3Rs is derived from their scattered expression in cortical areas [19]; apparently a few D3Rs mediate D3-specific actions in wide areas of the brain. While D2R mRNA is expressed in all major brain areas receiving dopaminergic projections, D3Rs are expressed in a more restricted manner (see first section). Despite the weak labeling observed in cortical areas, distinct population of fluorescently labeled D3R-positive pyramidal cells in the prefrontal cortex were clearly visualized [23] that could be due to the potential differences in sensitivity of signal detection.

There are various strategies available for the D3R system that may overcome the limitation arising from the low expression level of the receptor. DA has the highest affinity for D3R amongst all DA receptor subtypes [16,279] allowing a relatively small number of receptors to mediate significant cellular processes. Indeed, D3R activation was shown to inhibit calcium influx via low-voltage-activated Ca_v_3.2 calcium channels localized at the axon initial segment in prefrontal pyramidal cells [23] and auditory brainstem interneurons [280]. At this particular site of the neuron, only a few activated receptors can effectively influence downstream the ongoing activity of the cell by altering the generation of action potentials. Indeed, D3R activation leads to a marked suppression in the generation of high-frequency action potential bursts through Ca_v_3.2 calcium channels [23]. It seems that the axonal target site for D3Rs could be one key element by which D3Rs can multiply their effectiveness. In support of this notion, the effect of axonal D3Rs were visualized by 2-photon microscopy of axon varicosities of nucleus accumbens medium spiny neurons [281].

The increase in excitability of neurons can also be achieved in other ways by D3Rs. By the reduction of ongoing inhibitory activity—most typically mediated by GABA_A_ receptors in neurons—D3Rs can also increase excitability. In particular, D3Rs were found to reduce inhibitory synaptic inputs through the endocytosis of GABA_A_ receptors in the hippocampus [26] and in the nucleus accumbens [282,283]. This disinhibitory mechanism could be another key mechanism by which DA can influence the processing of neuronal signals in the hippocampal circuitry through D3R [284]. It remains speculative whether alteration of GABA_A_ function may stand behind the well-described effect of D3Rs on gamma oscillatory activity as a cellular-level mechanism. Corroborating this assumption, synchronous perisomatic inhibition by fast spiking interneurons is likely responsible for the generation of gamma oscillations, thus the reduction of the synchronizing currents by activated D3Rs could explain the gamma-inhibiting effects. Alternatively, it is also a possibility that D3Rs located in glutamatergic synapses may effectively influence excitatory transmission as shown in local neural networks of the nucleus accumbens [192].

### 8.3. Importance of the Non-Canonical Signaling in Local Functions

It is of particular interest that the D3R-mediated regulation of Ca_V_3 channels at the axon initial segment that leads to changes in excitability in auditory brainstem neurons occurs through a ß-arrestin-dependent mechanism [22]. The Ca-channel modulation at this site could also suppress burst firing in D3R-positive prefrontal neurons [23]. Cariprazine, as a D3R-preferring ligand in binding experiments, could also activate the ß-arrestin pathway as a partial agonist [285,286]. Taken together, it is assumed that the low-level ß-arrestin-dependent effect following D3R activation by cariprazine could be the mechanism by which this drug interacts with high-frequency oscillations in the hippocampus. The significance of non-canonical downstream signaling of D3Rs is further corroborated by the finding that D3R-induced reduction in GABA_A_ currents occurs through the endocytosis of GABA_A_ receptors [282].

### 8.4. Signs of D3R-Specific Regulation of Cellular Processes behind Cognitive Function

The activation of D3Rs can influence cellular-level processes of memory and learning as revealed by enhancement of long-term potentiation (LTP) by D3R activation in the CA1 region of the hippocampus in rats [287]. As LTP is considered as a cellular correlate of memory processing [288], this finding corroborates the idea that D3R modulation may improve cognitive performance in cognitive tests (see behavioral section). D3Rs maintain neuronal communication by the regulation of excitability especially in conditions when trains of action potentials are generated in the network. D3R neurons possess lower instantaneous spike frequency at train onset with slower action potential rise times [23]. On the other hand, the D3R agonist quinpirole can suppress evoked Ca influx in the axon initial segment [22] leading to decreased excitability in auditory interneurons. In contrast, application of the D3R agonist PD128907 increases the firing rate of medium spiny neurons in the nucleus accumbens, but not in prefrontal pyramidal neurons [282]. Therefore, full antagonists of D3Rs mediate complex effects on excitability depending on cell types and localization of receptors while partial agonists may balance train frequency to an optimal level.

## 9. Drugs with D3R Actions in the Therapy

Based on brain localization and pharmacological features of D3Rs, several possible therapeutic areas were presumed as potential clinical targets. Drugs behaving as D3R preferring agonists can have effective antiparkinsonian properties and reduce the symptoms of PD or the levodopa-induced dyskinesia in PD [289,290]. Some of these D3R preferring agonist drugs can be also effective for the treatment of depression [291] and RLS or periodic limb movements [292]. On the other hand, drugs showing antagonist or partial agonist properties with a wide variety of low to high selectivity at D3Rs can be effective for the treatment of schizophrenia [102,192], including predominant negative symptoms [293], drug addiction [104,294] and depression [108].

In spite of the great expectation that highly selective D3R antagonists/partial agonists can bring a new era in the antipsychotic development and in the treatment of the different symptoms of schizophrenia, the fact is that very few clinical data and therapeutic evidence can be found in the literature about such drugs. The completed clinical studies are mainly human PET studies or rarely published phase I trials, except a few other examples. For the characterization of D3R occupancy and selectivity in human brain and for the estimation of possible therapeutic doses, the [^11^C]-(+)-PHNO binding by region of interest was introduced. Human PHNO PET study is an efficient tool for the characterization of the occupancy of both D3Rs and D2Rs in the living human brain [163]. Of course, for this purpose the optimal solution would be to use highly selective D3R and D2R antagonist PET ligands but up to now, no such compounds have been described. Regarding successful drug developments of drugs acting on the D3Rs, only a couple of these resulted in market authorization, namely some D3R preferring agonist such as pramipexole and ropinirole in PD and the D3R preferring antagonist/partial agonist antipsychotic cariprazine in schizophrenia, bipolar mania and bipolar depression indications. 

A comprehensive overview of these drugs and the clinical developments are summarized and listed in a tabular format in Table 7.

### 9.1. Highly Selective D3R Antagonists

ABT-925 is a selective D3R antagonist with an approximately 100-fold in vitro selectivity for D3R versus D2R. Based on PET study data [320] tested in the dose range of 50 mg to 600 mg, [^11^C](+)-PHNO binding in the D3R rich regions were 90–100% (globus pallidus, SN) while only 53–55% (putamen, caudate) in the D2R rich regions. ABT-925 was developed as a potential atypical antipsychotic. In a phase II double-blind, randomized, placebo-controlled study [208] efficacy and safety of ABT-925 was tested in patients with acute exacerbation of schizophrenia. Patients were treated for 6 weeks with placebo, or 50 mg and 150 mg of ABT-925 once daily. No statistically significant improvement was observed with either dose of ABT-925 compared to placebo on the primary, Positive and Negative Syndrome Scale (PANSS) total score, and secondary efficacy endpoints. Ineffectiveness of the drug based on the above presented PET study data was explained by the authors with the not adequately selected (low) doses of the drug for the phase II study. 

GSK 598809 using [^11^C]-(+)-PHNO PET in human brains demonstrated dose-dependent occupancy of the D3 receptors as a competitive antagonist with more than 100-fold selectivity over D2Rs [295]. The drug was administered orally at doses of 5–175 mg. Single dose of GSK598809 administered to smokers was not affecting the rewarding properties of nicotine showing that it does not interfere with the primary reinforcing properties. On the other hand, it reduced the self-reported craving and slightly increased smoking. In further studies different types of smokers were investigated to understand if D3R antagonists decrease the rewarding properties of nicotine in humans. In a double-blind, placebo-controlled, parallel group trial, GSK598809 was added to cognitive behavioral therapy (CBT) and nicotine replacement therapy (NRT) for smoking cessation and prevention of very early relapse to smoking at a dose of 60 mg (NCT01188967). No conclusive results are available probably due to the low number (5 GSK 598809 treated) of completing subjects.

### 9.2. Dopamine D3R/D2R Antagonists with Preference for D3Rs

S33138 is a D3R preferring antagonist with about 25-fold in vitro selectivity for human D3R versus D2R [321]. The antipsychotic and anti-abuse efficacy of the drug was characterized in several animal models [322,323]. In a pilot human phase II study [296] the safety and efficacy profile of S33138 versus risperidone was investigated in patients with predominant positive symptoms of schizophrenia. Patients randomly received 5, 10, or 20 mg/day doses of S33138 or 4 mg/day dose of risperidone for 8 weeks. The largest improvement was seen with the 20 mg dose on the PANSS total score, but this was markedly lower than with risperidone. The mean decrease from baseline in PANSS negative score was higher with the 20 mg dose than with risperidone (−3.7 vs. −2.8). A second, phase IIb proof of efficacy trial has been also started [324] but this study was cancelled with no patients enrolled.

F17464 is a new, highly potent preferential D3R antagonist with 38–71-fold in vitro selectivity versus D2R for the treatment of schizophrenia [325]. In a phase II, double-blind, randomized, placebo-controlled, parallel-group efficacy and safety study of F17464, 20 mg twice daily, was evaluated versus placebo after 6 weeks treatment in patients with acute exacerbation of schizophrenia. The change from baseline of PANSS total score to Day 43 showed a statistically significant difference in favor of F17464 over placebo [141]. The difference became significant only after 3 weeks of treatment.

### 9.3. Dopamine D3R/D2R Partial Agonists with Preference for D3Rs

Bifeprunox is a D3R, D2R and 5-HT_1A_ receptor partial agonist with high affinity and about 4-fold selectivity for D3Rs over the D2R [326]. Clinical development of bifeprunox shows that bifeprunox has antipsychotic activity in placebo-controlled studies [297,298,299,300,301]. In the 6-week, double-blind, placebo-controlled studies patients were randomized to once-daily treatment with different fix doses of bifeprunox (5, 10, 20, 30 and 40 mg), placebo, or reference drugs (risperidone 6 mg, olanzapine 15 mg). Bifeprunox at 20 and 30 mg showed statistically significant improvement in PANSS total score compared to placebo. No statistically significant differences in efficacy were seen at the 5, 10 and 40 mg dose levels. In a 6-month phase III trial 20 and 30 mg/day dose of bifeprunox showed significantly longer time to deterioration compared to placebo. In 2007 bifeprunox schizophrenia NDA was filed to FDA, but it was rejected and after that the development of the drug was discontinued.

BP-897 is a high affinity, D3R antagonist/partial agonist with 70-fold selectivity for the D3R over the D2R [155]. It has mainly been tested for cocaine addiction in several animal models. BP-897 entered phase I study in 1999 with cocaine abuse and withdrawal indication, but its development was discontinued. In 2001 it entered phase II studies for the treatment of cocaine, nicotine and alcohol addiction, schizophrenia, and PD [302]. 

Cariprazine is the first registered D3R preferring antagonist/partial agonist drug with 6–8-fold in vitro selectivity over the D2R [327]. In 2015 cariprazine (VRAYLAR^®^) was approved in the United States for the treatment of schizophrenia (1.5 to 6 mg/day) in adults and in 2017 as Reagila^®^ in the European Union. In 2015 it was also registered for the treatment of acute manic or mixed episodes (3 to 6 mg/day) and in 2019 for the treatment of depressive episodes (bipolar depression; 1.5 to 3 mg/day) associated with bipolar I disorder by the FDA. In a human PET study to separately assess the D3R and D2R occupancies by the D3R-preferring D3R/D2R agonist PET-ligand PHNO, near complete receptor occupancy at both D2R and D3R subtypes was observed after 15 days of dosing at 12 mg/day [328]. At the lowest dose of 1 mg/day, mean D3R and D2R occupancy was 76% and 45%, while at the 3 mg/day dose it was 92% and 79%, respectively. These occupancy data first provided evidence that cariprazine is an antipsychotic which dose dependently occupies both the D2R and D3R receptors not only in vitro but also in vivo with an 3.5–5.5-fold selectivity toward the D3R over the D2R. In the treatment of patients with schizophrenia, cariprazine demonstrated efficacy in 3 phase II/III 6-week, randomized, double-blind, placebo-controlled trials in the dose range of 1.5 to 9 mg/day [303,304,305]. In each of the 3 trials, a statistically significant and clinically meaningful improvement was seen for cariprazine versus placebo in both the primary and secondary efficacy parameters, i.e., change from baseline to end of Week 6 in the PANSS total score and Clinical Global Impressions-Severity scale (CGI-S). In all three trials improvement was dose-dependent, so higher doses resulted greater reductions in symptoms. Long-term efficacy of cariprazine was investigated in the dose range of 3 to 9 mg/day in a relapse prevention study [306]. Cariprazine significantly delayed the time to relapse of schizophrenia compared to placebo. The percentage of placebo treated patients, who relapsed (47.5%) was almost double than that of cariprazine treated subjects (24.8%). Pharmacodynamic properties, as well as affinity for the D3R and serotonin 5-HT_1A_ receptor provided a rationale to investigate cariprazine as a monotherapy in the treatment of patients with predominant negative symptoms (PNS) of schizophrenia. Patients with PNS, measured by PANSS negative factor score (PANSS-NFS) and patient functioning, measured by Personal and Social Performance (PSP) scale improved significantly better after six months of treatment in the cariprazine treatment group (dose range of 3 to 6 mg/day with a target dose of 4.5 mg/day) than in the risperidone treatment group (dose range of 3 to 6 mg/day with a target dose of 4 mg/day) [293]. The robustness of the study results was confirmed by the exclusion of pseudospecific effects, which could have contributed to the positive results. Based on post hoc analyses, the cognitive functioning of patients also showed significant improvement [207]. In the treatment of patients with manic or mixed episodes associated with bipolar I disorder, cariprazine also demonstrated efficacy in 3 phase II/III, randomized, double-blind, placebo-controlled 3-week studies. The investigated dose range of cariprazine in these studies was 3–12 mg/day [304,307,308]. In all of these studies Young Mania Rating Scale (YMRS) total score as primary and CGI-S as key secondary endpoints were assessed. In each of the 3 trials, statistically significant and clinically meaningful improvement was seen for cariprazine relative to placebo in both the primary and secondary efficacy parameters. Two different types of depression indication were also examined, namely bipolar depression and major depressive disorder (MDD) as an adjunctive therapy. In bipolar depression 3 phase II/III, 6-week, randomized, double-blind, placebo-controlled studies were carried out [310,311,312]. A statistically significant improvement in favor of cariprazine 1.5 and 3 mg/day versus placebo was seen on the Montgomery Åsberg Depression Rating Scale (MADRS) total score and CGI-S change from baseline. In the first proof-of-concept trial in MDD the 2–4.5 mg/day cariprazine dose was superior to placebo as an adjunctive treatment to standard antidepressant therapy in terms of change of MADRS total score and response rates [313]. In this indication further pivotal studies are in progress. A recently published case report provided the first evidence that cariprazine showed beneficial effect not only in the reduction of bipolar depression symptoms but also a marked reduction in alcohol or cannabis dependence, achieving abstinence after longer term treatment [329].

### 9.4. Dopamine D3R/D2R Agonists with Preference for D3Rs

Pramipexole is the most widely used D3R-preferring DA agonist with about 8-fold selectivity over the D2R [330] for the treatment of motor symptoms at all stages of PD [290,291]. This has a special importance because although L-DOPA is the gold standard therapy for reducing the motor symptoms of PD, but during long-term use motor complications and drug-induced dyskinesias appear affecting about one-third of patients after as early as only 2 years of L-DOPA treatment. In advanced PD patients 3 double-blind, placebo-controlled trials were conducted with pramipexole [331]. One additional trial was conducted where in addition to pramipexole, bromocriptine was also compared with placebo. Two more studies with early PD [331] were also conducted. Early disease was defined as <7 years duration and characterized as stage I–III on the Modified Hoehn and Yahr scale, while advanced disease was characterized as stage II–IV on the same scale. Advanced disease patients were taking L-DOPA, and in all studies the Unified Parkinson’s Disease Rating Scale (UPDRS) was used as primary efficacy measure. The total UPDRS score was significantly improved in all pramipexole groups (dose ranged from 0.26 to 4.6 mg/day) compared to placebo. In the comparative trial pramipexole and bromocriptine were superior to placebo and pramipexole efficacy was comparable with bromocriptine treatment in advanced PD. Similarly, in the early PD trials pramipexole showed statistically significant decreases in primary outcome measure compared to placebo [332]. Pramipexole was also tested in major depression with or without melancholia and without psychotic features. Three different daily doses of pramipexol (0.375 mg, 1.0 mg, and 5.0 mg) were compared to fluoxetine (20 mg) and placebo in a randomized, double-blind, parallel-group study [314]. After 8 weeks treatment patients receiving 1.0 mg/d dose pramipexole showed significant improvement over baseline compared to placebo by measures of Hamilton Psychiatric Rating Scale for Depression (HAM-D), the MADRS, and the CGI-S scale. Marked improvement was seen at the 5.0 mg/day dose level, but a substantial dropout rate increase was seen compared to fluoxetine. The lowest dose (0.375 mg/day) of pramipexol was not different from placebo. The efficacy of pramipexole has also been evaluated in RLS versus placebo [315]. The dose was individually set in a range of 0.125–0.75 mg/day. Efficacy was measured by the International RLS Study Group Rating Scale (IRLS) and CGI-I. After 6 weeks of treatment pramipexole showed highly significant improvement compared to placebo.

Ropinirole is a D3R preferring DA agonist with 10-fold selectivity over the D2R [333]. Its efficacy in PD was proven in a couple of controlled trials in monotherapy and also in combination treatment. Ropinirole has also been approved for the treatment of RLS [316]. Ropinirole was tested for the treatment of early PD classified in disease stages I to III according to Hoehn and Yahr in a prospective, randomized, double-blind and placebo-controlled six-month study [334]. The primary endpoint of the study was the motor score of UPDRS and while ropinirole patients improved, the placebo group deteriorated. The responder rate for ropinirole was more than twice as high than for placebo. The maximum applied dose was 24 mg/day. A further study was conducted with ropinirole in the late stage of PD, where less L-DOPA treatment was found to be needed compared to placebo [335]. Ropinirole is the most widely investigated DA receptor agonist in RLS. It has market authorization for the treatment of RLS in several countries. Its efficacy on RLS has been confirmed in many controlled studies [316]. Prolonged release formulation for once daily treatment of pramipexole and ropinirole has been developed and confirmed [336,337].

Rotigotine is a non-ergoline DA receptor agonist with 19-fold selectivity for the D3R over the D2R [338]. Transdermal rotigotine dose-dependently improved the UPDRS, Activities of Daily Living and motor subtotal scores in patients with early-stage PD. The antiparkinsonian effect was significant compared to placebo [317]. Transdermal rotigotine also improved the symptoms of RLS in two six-month trials in adults with idiopathic, moderate to severe RLS. Rotigotine significantly (1–3 mg/day) decreased the IRLS total score [318,319].

## 10. Conclusions and Future Directions

D3Rs were discovered 30 years ago. Since then, it has been shown that these receptors are implicated in several CNS functions such as movement control, reward, feeding, olfaction, learning and cognition. It has been recognized that D3Rs may also be involved in several psychiatric (e.g., schizophrenia, depression, anxiety etc.) and neurodegenerative diseases (e.g., PD). Consequently, D3Rs have become promising targets for drug research and great efforts have been made to develop D3R selective and high affinity ligands useful for basic research and to obtain compounds with therapeutic utility. 

This review made an attempt to overview several aspects of D3R research including the anatomical distribution of this receptor in the brain and periphery, molecular biology, the related drug research, its roles in behaviors and at the brain network level. An overview was given on the therapeutic utility of drugs with D3R affinities and various functionalities and how the results have been translated into therapy of various CNS diseases. 

D2Rs and D3Rs show high degree of similarity in molecular structure, cellular functions and signalization which greatly hampered development of selective D3R ligands. An emerging number of experimental structures in the D2-like receptor family have been a great aid to differentiate the molecular structure of the D3R and D2R. Exploration of subtle differences in the molecular structure, the better understanding of the nature of receptor-ligand binding modes and their relation to functionality considerably increased our opportunities to design new ligands specifically targeting both orthosteric and allosteric binding sites (i.e., bitopic ligands) and obtaining compounds with high affinity, more selectivity and targeted biased functionality.

Regional distribution of D3Rs within the brain and in the periphery of experimental animals and in human has largely been explored. Current research should be directed to better understand the subregional, cellular, and subcellular localization of D3Rs in more detail and their roles in regulating local and network functions. 

Beyond its direct signaling effects, the modulatory role of D3R in cellular signal transduction is yet to be explored. Via supramolecular receptorial complexes as well as targeted signaling at the axon initial segment, D3R might effectively tune other neurotransmitter pathways in neurons. The functional consequences and modulatory potential in these interactions in situ are yet to be assessed using advanced molecular techniques.

Exploration of the specific roles/functions of a given receptor obviously requires the availability of appropriate tools, i.e., selective, high affinity ligands with different functionality (agonism, antagonism or partial agonism) having favorable physicochemical and pharmacokinetic properties. The lack of such “ideal” pharmacological tools delayed the assessment of the exact role(s) of D3R in preclinical pharmacological studies. In fact, great number of D3R agonists have been published which showed high affinity and high selectivity under in vitro conditions. However, a specific problem related to agonist testing—less prevalent in case of antagonists—has always been that the affinity and selectivity data as well as the compounds’ functional profile across various laboratories using different assay conditions proved to be highly variable. The apparent inconsistencies in affinity, selectivity and functional data thus have made it difficult to assess the usability of the published compounds. However, as we brought forward some examples, efforts are still ongoing to overcome such difficulties. A better understanding of the molecular structure and signaling properties of D3Rs will offer a great help to obtain high affinity and selectivity with designed functional profile. The development of high affinity, truly high selectivity agonists and antagonist for D3Rs with good pharmacokinetic properties are still required for fundamental, and especially for in vivo research. Current activity to develop high affinity, selective D3R ligands (either agonists or antagonists) of clinical utility has greatly ceased. 

The discovery and application of D3R preferring D3R/D2R agonist [^11^C](+)-PHNO as PET ligand significantly enhanced our understanding through the visualization/imaging of D3R and its in vivo occupancy following drug treatment. Obviously, [^11^C](+)-PHNO is not an ideal PET ligand, and efforts are also continuing to obtain PET ligands (agonist? antagonist?) with better selectivity for D3Rs and favorable pharmacokinetic properties. 

Although a lot of knowledge has been amassed since the discovery of the D3R, the precise role of this receptor in the regulation of animal behaviors including locomotion, cognition and socio-emotionality is still somewhat elusive. The lack of a true breakthrough in this respect is at least partly related to the limited in vivo selectivity of available pharmacological tools. A better apprehension of the role that D3Rs play in the regulation of the above behaviors depends on the emergence of in vivo substantially selective receptor ligands and the employment of state-of-the-art technologies such as DREADD, conditional knock-outs and optogenetic approaches.

After recognition that several antipsychotics display considerable affinity for D3Rs in vitro, there was a great expectation that D3R antagonists/partial agonists with high affinity and selectivity can bring a new era in schizophrenia and drug abuse therapy. Selective D3R antagonists did, in fact showed D3R occupancy in the brain but their clinical efficacy fell short of expectations. For the time being, there exist no D3R selective antagonists/partial agonists in the therapeutic armamentarium. On the other hand, compounds with a multi-receptorial profile and significant D3R affinity have become important therapeutic tools. Drugs behaving as D3R preferring agonists potently reduce the symptoms of PD or the levodopa-induced dyskinesia in PD. Some of these D3R preferring agonists can effectively be used also for the treatment of depression, RLS or periodic limb movement. The high affinity D3R preferring D3R/D2R antagonist/partial agonist cariprazine is an example of a potent atypical antipsychotic in the treatment of schizophrenia including primary negative symptoms, bipolar mania and bipolar depression indications. 

In conclusion, in the last 30 years that elapsed since the discovery of D3Rs we have learned a great deal about its structure, signaling properties and functions in behavior. Research for a better understanding of the roles of D3R and development of selective ligands for D3Rs is ongoing. These efforts are important for basic as well as clinical research. Although D3R selective antagonists/agonists have not yet become therapeutically useful drugs, their application as add-on therapy could be an option. On the other hand, compounds with multi-receptorial profile displaying high affinity for D3R (agonists, partial agonists and antagonists) have been shown to be valuable drugs for the treatment of psychiatric diseases and PD. Designing and testing strategy of these multi-receptorial compounds with high affinity for D3Rs obviously requires complex and fine-tuned approaches different from those used for a single target.

## Figures and Tables

**Table 1:** Percent identity matrix of D_2_-like receptors of different species created by Clustal2.1 software [64]; h: human; r: rat; m: mouse.

Name	UniProt ID	hD2R	hD3R	rD3R	mD3R	hD4R
hD2R (long)	P14416-1	100.0	58.5	54.1	54.1	38.4
hD3R	P35462-1	58.5	100.0	89.0	90.5	40.7
rD3R	P19020-1	54.1	89.0	100.0	97.1	40.3
mD3R (long)	P30728-1	54.1	90.5	97.1	100.0	39.8
hD4R	P21917-1	38.4	40.7	40.3	39.8	100.0

**Table 2:** Cognitive effects of D3R selective antagonists.

Compound	Behavioral Assay	Impairing Agent	Species	Effect	References
SB-277011	water labyrinth	FG-7142; scopolamine	rat	+	[194]
	social recognition memory	scopolamine	rat	+	[182]
	social recognition memory	delay	rat	+	[179]
	novel object recognition	scopolamine	rat	+	[193]
	active shock avoidance		rat	Ø	[193]
	Morris water maze	MK-801	mouse	Ø	[199]
	novel object recognition	chronic mild stress	rat	+	[195]
RGH-1756	water labyrinth	FG-7142; scopolamine	rat	+	[194]
S33084	social recognition memory	scopolamine	rat	+	[182]
	social recognition memory	delay	rat	+	[179,182]
	novel object recognition	social isolation	rat	+	[196]
	novel object recognition	delay	rat	+	[185]
	social novelty discrimination	delay	rat	+	[185]
S14297	social recognition memory	scopolamine	rat	+	[182]
	social recognition memory	delay	rat	+	[182]
Y-QA31	novel object recognition	MK-801	mouse	+	[197]
FAUC365	novel object recognition	DA transporter knockdown	mouse	+	[202]

+: improving effect, Ø: lack of efficacy.

**Table 3:** Cognitive effects of D3R preferring, selective D3R/D2R antagonists.

Compound	Behavioral Assay	Impairing Agent	Species	Effect	References
S33138	novel object recognition	delay	rat	+	[201]
	attentional set-shifting	MPTP	Rhesus macaque	+	[201]
	variable delayed-response task	MPTP	Rhesus macaque	+	[201]
	delayed matching to sample	age	Rhesus macaque	+	[201]
	novel object recognition	social isolation	rat	+	[196]
U99194	water labyrinth	FG-7142; scopolamine	rat	+	[194]
	active shock avoidance	-	rat	Ø	[193]
	novel object recognition	scopolamine	rat	+	[193]
	novel object recognition	chronic WIN55,212-2 in adolescence	rat	+	[203]
RG-15	water labyrinth	scopolamine	rat	+	[136]
F17464	5-choice serial reaction time test	-	rat	Ø	[200]
	Morris water maze	-	rat	Ø	[200]
nafadotride	passive avoidance	-	rat	Ø	[198]
	novel object recognition	delay	rat	Ø	[198]
	8-arm maze	-	rat	Ø	[198]

+: improving effect, Ø: lack of efficacy, -: no impairing agent applied.

**Table 4:** Cognitive effects of D3R preferring partial agonists.

Compound	Behavioral Assay	Impairing Agent	Species	Effect	References
BP-897	water labyrinth	FG-7142; scopolamine	rat	+	[194]
cariprazine	water labyrinth	scopolamine	rat	+	[150]
	T-maze	PCP	mouse	+	[209]
	attentional set-shifting	PCP	mouse	+	[209]
	social recognition memory	PCP	mouse	+	[209]
	novel object recognition	delay	rat	+	[210]
	novel object recognition	neonatal PCP, social isolation	rat	+	[210]
	novel object recognition	PCP	rat	+	[205]
	operant reversal learning	PCP	rat	+	[205]
	5-choice serial reaction time test	PCP	rat	+	[206]

+; improving effect.

**Table 5:** Behavioral effects of D3R ligands on anxiety, anhedonia and stress reactivity.

Compound	Behavioral Assay	Impairing Agent	Species	Effect	References
nafadotride	conflict drinking (Vogel)	-	rat	+	[222]
	four plate	-	mouse	Ø	[222]
U99194	conflict drinking (Vogel)	-	rat	+	[222]
	four plate	-	mouse	+	[222]
	light-dark box	-	rat	+	[223]
	elevated plus maze	-	rat	+	[223]
	open field	chronic WIN55,212–2	rat	Ø	[203]
BP-897	conflict drinking (Vogel)	-	rat	+	[227]
	forced swimming	-	rat	Ø	[228]
cariprazine	sucrose consumption	chronic unpredictable stress	mouse	+	[229]
	novelty-induced hypophagia	chronic unpredictable stress	mouse	+	[229]
	sucrose consumption	chronic mild stress	rat	+	[230]
7-OH-DPAT	conflict drinking (Vogel)	-	rat	+	[227]
	elevated plus maze	-	rat	+	[228]
	forced swimming	-	rat	+	[228]
	elevated plus maze	-	mouse	+	[225]
	tail suspension	-	mouse	+	[224]
ropinirole	forced swimming	-	mouse	+	[127]
	forced swimming	-	rat	+	[127]
	marble bury	-	mouse	+	[127]
	aggression	social isolation	mouse	+	[127]
	ultrasonic vocalization	aversive environment	rat	+	[127]
	conflict drinking (Vogel)	-	rat	Ø	[127]
	elevated plus maze	-	rat	Ø	[127]
S32504	forced swimming	-	mouse	+	[127]
	forced swimming	-	rat	+	[127]
	learned helplessness	-	rat	+	[127]
	sucrose consumption	chronic mild stress	rat	+	[127]
	marble bury	-	mouse	+	[127]
	aggression	social isolation	mouse	+	[127]
	ultrasonic vocalization	aversive environment	rat	+	[127]
	conflict drinking (Vogel)	-	rat	+	[127]
	elevated plus maze	-	rat	Ø	[127]

+: improving effect, Ø: lack of efficacy, -: no impairing agent applied.

**Table 6:** Social behavioral effects of D3 ligands.

Compound	Behavioral Assay	Impairing Agent	Species	Effect	References
A-690344	huddling	PD-128907	rat	+	[233]
ABT-127	huddling	PD-128907	rat	+	[234]
ABT-925	huddling	PD-128907	rat	+	[235]
U99194	social interaction	-	mouse	+	[236]
	social interaction	PCP	mouse	+	[154]
F17141	social interaction	MK-801	mouse	+	[192]
cariprazine	social interaction	PCP	rat	+	[205]
	social interaction	neonatal PCP, social isolation	rat	+	[210]
	social interaction	PCP	mouse	+	[209]

+: improving effect, -: no impairing agent applied.

**Table 7:** Overview of clinical trials on the D3R acting drugs.

Drugs	Selectivity	Indication	Study Outcomes	References
**Highly selective D3R antagonists**				
ABT-925	100-fold (in vitro)	schizophrenia	no significant improvement on PANSS total	[208]
GSK 598809	>100-fold (in vivo, human *PET*)	drug abuse	not affecting the rewarding properties of nicotine	[295]
		drug abuse	add-on to CB and NR therapies without conclusive results	NCT01188967
**D3R preferring D3R/D2R antagonists**				
S33138	~25-fold (human in vitro)	schizophrenia cocaine abuse	phase IIa study in patients with predominant positive symptoms of schizophrenia with markedly lower improvement vs. risperidone	[296]
F 17464	38–71-fold (in vitro human)	schizophrenia	phase II trial in acute patient with schizophrenia with significant improvement	[141]
**D3R preferring D3R/D2R partial agonists**				
bifeprunox	4-fold	schizophrenia	20 and 30 mg showed significant improvement both in the short and long term, prolongation of time to deterioration, treatment, while not significant improvements were seen at doses 5, 10 and 40 mg.	[297,298,299,300,301]
BP-897	70-fold	drug abuse schizophrenia PD		[302]
cariprazine	6–8-fold (in vitro human) 3.5–5.5 (in vivo human)	schizophrenia	significant improvement both in the short and long term, relapse prevention studies, at each investigated dose levels compared to placebo therapeutic dose range: 1.5–6 mg/d	[303,304,305,306]
		predominant negative symptoms of schizophrenia	significantly higher improvement both in the symptoms and functionality of patient compared to risperidone	[293]
		bipolar mania	significantly higher improvement in acute manic patients after cariprazine compared to placebo treatment therapeutic dose range: 3–6 mg/d	[307,308,309]
		bipolar depression	significant improvement compared to placebo therapeutic dose range: 1.5–3 mg/d	[310,311,312]
		major depression add-on	2–4.5 mg/d cariprazine dose is superior to placebo as an adjunctive therapy to standard antidepressant treatment	[313]
**D3R preferring D3R/D2R agonists**				
pramipexole	8-fold	motor symptoms at all stages of PD	total UPDRS score was significantly improved compared to placebo. in early PD pramipexole also showed significant decrease of symptoms	[290,291]
		depression	1.0 mg/d dose showed significant improvement compared to placebo. At higher dose (5 mg/d) the dropout rate increased markedly.	[314]
		RLS	after 6 weeks treatment highly significant improvement compared to placebo measured by the IRLS	[315]
ropinirole	10-fold	PD	significant improvement was demonstrated in the early and late stage of PD	[316]
		RLS	low dose (0.25 to 6 mg) showed significant improvement on IRLS compared to placebo	[316]
rotigotine (patch)	19-fold	PD	dose-dependently improved the UPDRS in patients with early-stage PD	[317]
		RLS	significantly decreased the IRLS total score in the 1–3 mg/d dose range	[318,319]

## Data Availability

Data sharing not applicable.
